# Offspring's exposome: a narrative review on the influence of early-life factors on childhood obesity risk

**DOI:** 10.3389/fnut.2025.1597746

**Published:** 2025-10-06

**Authors:** Beatrice Maccarini, Federica Loperfido, Irene Bianco, Francesca Sottotetti, Dana El Masri, Chiara Ferrara, Federica Verme, Erika Cangelosi, Niccolò Meriggi, Carlotta De Filippo, Hellas Cena, Rachele De Giuseppe

**Affiliations:** ^1^Laboratory of Dietetics and Clinical Nutrition, Department of Public Health, Experimental and Forensic Medicine, University of Pavia, Pavia, Italy; ^2^Institute of Agricultural Biology and Biotechnology (IBBA), National Research Council (CNR), Pisa, Italy; ^3^Clinical Nutrition and Dietetics Service, Unit of Internal Medicine and Endocrinology, ICS Maugeri IRCCS, Pavia, Italy

**Keywords:** childhood obesity, infant nutrition, exposome, gut microbiota, maternal lifestyle, human breast milk

## Abstract

Childhood obesity has emerged as a global health challenge, with significant long-term health consequences, including an increased risk of non-communicable diseases. The “first 1,000 days” period of life is a critical window for shaping long-term health outcomes. This narrative review aims to explore the role of environmental exposures, categorized within the exposome framework, in developing childhood obesity. The exposome encompasses three domains: general external exposures (e.g., air pollution, urbanization), specific external exposures [e.g., nutrition, physical activity, socioeconomic status (SES)], and internal exposures (e.g., metabolic responses, oxidative stress). Evidence identifies risk factors such as maternal smoking during pregnancy, early-life exposure to endocrine-disrupting chemicals, and air pollution, which contribute to obesogenic processes. In contrast, protective factors include access to green and blue spaces, exclusive breastfeeding, adequate complementary feeding, regular physical activity, limited screen time, and sufficient sleep, which support healthy growth trajectories. Findings regarding SES, perfluoroalkyl and polyfluoroalkyl substances exposure, and human breast milk macronutrient composition remain heterogeneous and context-dependent. The findings highlight the need to integrate public health strategies addressing modifiable environmental and lifestyle factors. Identifying a “healthy exposome” that protects against obesity risk can steer the development of personalized prevention strategies, ultimately reducing the burden of obesity and associated diseases.

## 1 Introduction

The prevalence of childhood obesity has risen alarmingly worldwide. In 2022, 159 million children and adolescents were affected by obesity, a rate four times higher than in 1990 (1). Childhood obesity is strongly associated with an increased risk of early onset of other non-communicable diseases (NCDs), including type 2 diabetes, cardiovascular diseases, and certain cancers, leading to adverse health, economic, and social consequences (2, 3).

The developmental origins of health and disease (DOHaD) hypothesis suggests that environmental exposures during critical periods of early life influence disease risk by altering biological pathways related to metabolism, inflammation, and energy homeostasis (4). Preventing childhood obesity has become a global public health challenge, giving attention to modifiable exposure factors during critical developmental periods, such as the prenatal and early childhood stages (5). In particular, the “first 1,000 days” period, from conception to the child's second year of age, represents a crucial window for shaping long-term development and health outcomes (6, 7).

Particularly, maternal factors, including nutrition, stress, and exposure to environmental pollutants, can influence fetal programming, shaping the development of the child's immune system, metabolic pathways, and brain function (8). These exposures may induce epigenetic changes and alter gene expression, impacting long-term health outcomes. The interplay between maternal exposures and early-life environment highlights the pivotal role of the “first 1,000 days” in determining lifelong health trajectories (7, 9).

Consequently, there is an increasing need to investigate the complex totality of external and internal exposures that affect the risk of obesity and NCDs from conception onward (10). Measuring the interaction of different exposures throughout life is highly complicated and challenging. In this context, the exposome concept was introduced in 2005, defined as the totality of environmental factors that potentially influence human health across the lifespan (11). The “exposome approach” represents a novel perspective aimed at moving beyond the study of the relationship between individual environmental factors and health outcomes, by integrating multiple risk factors and examining their interactions and potential causal mechanisms related to various health outcomes (11).

The exposome is divided into three different domains: (i) general external exposome, encompassing factors such as social capital, urbanization, air pollution, and climate; (ii) specific external exposome, which includes aspects such as nutrition, physical activity (PA), and other lifestyle habits, as well as social-economic determinants; and (iii) internal exposome, which involves endogenous biological responses unique to everyone, including metabolic factors, oxidative stress, inflammation, circulating blood biomarkers, hormones, and microbiome (12).

In the context of the “first 1,000 days” of life, the present review will provide an in-depth analysis of the infant exposome, categorized according to the domains previously described and adapted to the following research question.

Specifically, the review aims to identify exposure determinants associated with an increased risk of obesity during childhood and to define the characteristics and components of a “healthy exposome” in early life. The identification of protective factors may contribute to the development of effective preventive strategies for childhood obesity.

## 2 General external exposome and childhood obesity

Environmental pollutants, metals, chemicals, such as endocrine-disrupting chemicals (EDCs), and urbanization are components of the general external exposome (12). Recently, research has analyzed both prenatal and postnatal exposure to environmental factors, focusing on their influence on a higher risk of developing chronic diseases, including obesity.

### 2.1 Endocrine disrupting chemicals

Exposure to EDCs during early life seems to have an impact on the development of obesity, as shown in several original studies as well as systematic reviews (13).

EDCs include compounds such as bisphenol A (BPA), phthalates, and perfluoroalkyl and polyfluoroalkyl substances (PFAS). EDCs primarily originate from industrial processes and can be found in everyday settings, other than pesticides, clothing additives, toys, food items (e.g., beverages, cereals, canned food, and drinks, or labeled fruit), and packaging materials, including ultra-processed food (13, 14). These compounds can disrupt the endocrine system's function, affecting organs such as the liver, pancreas, and reproductive system, potentially leading to various health issues, including neurodevelopmental and metabolic disorders like obesity (13). Particularly, phthalates and BPA have potential obesogenic effects, especially in vulnerable populations, including infants (15). Gutiérrez-Torres and colleagues investigated whether exposure to EDCs during the prenatal period may affect anthropometric variables and biochemical parameters in preschool-age children (ages 3-5) (16). Positive associations have been found in (i) percentage of fat mass, (ii) body mass index (BMI), (iii) waist circumference, and (iv) skinfolds. Furthermore, the risk of being overweight persisted after adjustment for key confounding variables (e.g., maternal BMI, birth weight, breastfeeding, sex of the child, smoking, and other environmental exposures). No association was detected between prenatal exposure and lipid profile or glucose levels in childhood (16).

In contrast, a recent systematic review and meta-analysis of 13 studies found no statistical association between prenatal exposure to four different PFAS compounds and BMI fluctuations or waist circumference in children aged 18 months to 11 years. Notably, the authors highlight that these results may be influenced by the timing of exposure and individual vulnerability (17).

These findings were in line with the results of Lin and colleagues, which reported no significant associations between prenatal BPA exposure and birth weight, birth length, or head circumference (18).

Symeonides and colleagues (19) conducted an umbrella review on both prenatal and postnatal exposures to various chemicals and their adverse effects on children's health. They found that exposure to BPA was linked to insulin resistance, obesity, and hypertension. Specifically, phthalate compounds were associated with insulin resistance, elevated blood pressure, and precocious puberty in girls. Furthermore, exposure to PFAS was related to an increased BMI and overweight status (19).

Research on postnatal exposure primarily focuses on children aged 3–19 years. Notably, Ribeiro et al. (20) found positive associations between EDCs exposure and several indicators of overweight or obesity, including BMI and waist circumference, in children (aged between 6 and 19 years). In particular, the meta-analysis highlighted a correlation between child exposure to 2,5-dichlorophenol (2,5-DCP) and obesity (OR = 1.8; CI: 1.1018, 3.184) (20). However, since most of the results are from observational studies, causality couldn't be definitively established (20).

Recent evidence (21) has also identified a positive correlation between exposure to several phthalate acid ester compounds (PAEs) and childhood obesity. Studies included were conducted on children and adolescents aged 3-19 worldwide, including the United States, China, Iran, South Korea, and Sweden. Strong associations were found throughout subgroup analysis between several phthalate metabolites in urinary samples and childhood obesity, especially in Asia (21). However, the heterogeneity of the upper tolerable values established by government authorities worldwide must be considered.

### 2.2 Air pollution

Current literature on air pollution has examined the risk of developing obesity, with a focus on exposures during early childhood and adulthood (22–24). Air pollutants mainly originate from combustion processes, including vehicular emissions, industrial activities, and the burning of fossil fuels (23). Although the mechanism connecting air pollution to a higher risk of obesity is not completely understood, biochemical processes are widely recognized as primary contributors. When air pollutants enter the body through the respiratory system, they can enhance oxidative stress levels in several tissues. Consequently, the inflammatory response may result in vascular damage and insulin resistance, affecting body weight (25). Prenatal exposure to air pollution, notably fine particulate matter (PM) with a diameter of ≤ 10 mm (PM_10_) and PM with a diameter of ≤ 2,5 mm (PM_2.5_), has been related to fetal growth restriction (26, 27). Young adults born with fetal growth restriction (FGR) are at increased risk of experiencing high blood pressure, reduced kidney function, hypertension, and cardiovascular complications later in life (28). However, the effects related to PM exposure on postnatal growth and childhood obesity remain unclear (29). Shao and colleagues investigated the impact of PM prenatal exposure on fetal development and its potential long-term health consequences, showing that prenatal exposure to PM_2, 5_, PM_10_, sulfur dioxide (SO_2_), and ozone (O_3_) was significantly associated with reduced fetal biometry at 24 weeks of gestation (GW), with SO_2_ having the most pronounced effect (26). At GW36, exposure to air pollution continued to negatively affect fetal size, although the effects were less significant compared to the earlier stage of pregnancy. Fetuses in the highest exposure quartile registered intrauterine weights that were 6.3% lower at GW24 and 2.1% lower at GW36 than those in the lowest quartile (26). However, no significant difference in birth weight was observed, suggesting that rapid growth occurred during the third trimester to offset earlier growth restrictions (30). Mergetaki et al. (29) examined the association between prenatal air pollution exposure and obesity-related parameters in children by analyzing data from 633 mother-child pairs. Prenatal exposure to PM was not associated with adiposity at 4 years of age. However, increased prenatal exposure to PM_2.5_ and PM_10_ was linked to a higher risk of obesity (OR = 1.15; 95% CI: 1.01–1.31, *p* = 0.04) and abdominal obesity (OR = 1.18; 95% CI: 1.03–1.35, *p* = 0.03) at 6 years, respectively (29).

Recently, Zheng et al. confirmed findings from previous research in their systematic review, emphasizing several key factors (e.g., duration of exposure, geographic region, country's level of development) that influence the relationship between exposure to air pollution and childhood excessive weight. The study found that short-term exposure (<1 year) and long-term exposure (1 year or more) to PM_2.5_ had different effects on the risk of being overweight and obesity, with an OR of 1.18 (95% CI: 1.09, 1.27) (24).

Considering geographic differences, PM_2.5_ exposure significantly increased the risk of being overweight and obese in Asia, with an OR of 1.19 (95% CI: 1.10, 1.28). However, studies conducted in America and Europe did not find significant results. Moreover, when assessing the risk of overweight or obesity, developing countries exhibited a higher risk than developed countries for all pollutants considered (24).

Among the various pollutants examined, PM_1_ showed a significant negative impact on the development of overweight/obesity and BMI increase (24).

### 2.3 Urbanization

A further significant component of the general external exposome is urbanization, a global phenomenon that involves population growth and densification in urban areas (31). The extension of developed areas leads to greater environmental challenges, such as increased traffic congestion, higher levels of air and noise pollution, exacerbation of the urban heat island effect, and the depletion of accessible green and blue spaces (31). In this context, several systematic reviews investigated the role of Nature-based Solutions (NbS) on human health (32).

Currently, scientific literature lacks systematic reviews and meta-analyses that specifically address the role of prenatal exposure to green spaces. Consequently, our analysis draws upon alternative study designs. Heo et al. conducted a prospective cohort study in New York City, examining the effect of residential green space exposure on birth outcomes such as preterm birth (PTB), birth weight, and estimated fetal weight (EFW) (33). They found that although green space exposure did not significantly affect birth weight or EFW, greater exposure was associated with a reduced risk of PTB, suggesting potential benefits for fetal maturity and neonatal health beginning from the gestational period (33). Similarly, Toda et al., in an analysis of 11 European birth cohorts, reported that increased residential green space exposure was linked to higher birth weight and lower odds of being small for gestational age (SGA), with increased effects observed in more deprived populations (34). Furthermore, Marteines and colleagues explored the consequences of prenatal environmental exposures on BMI from birth to 24 months, finding that access to green spaces during pregnancy was associated with lower BMI *z*-scores at 24 months in a cohort of predominantly lower socioeconomic status (SES) participants (35).

Concerning postnatal exposure, a systematic review and meta-analysis showed that living in rural areas is associated with a higher prevalence of childhood obesity compared to children residing in urban areas of the United States. The meta-analysis (*n* = 74,168 participants aged 2–19) found that rural children have 26% higher odds of living with obesity compared to their urban counterparts (OR = 1.26; 95% CI: 1.21-1.32) (36).

The authors highlighted the obesity disparity between urban and rural children; however, the mechanisms driving these differences remain unclear (36). Previously Dunton and colleagues have documented comparable results, emphasizing the association between neighborhood characteristics, urban sprawl, and obesity outcomes among adolescents. Specifically, adolescents residing in rural, exurban, and mixed urban areas exhibited a higher likelihood of being overweight compared to their counterparts living in newer suburban, older suburban, and inner-city regions (37).

In contrast, recent evidence suggests that residing in urban areas is a significant risk factor for developing obesity. Specifically, children living in urban regions, particularly in the southern and northern areas of Mexico, registered higher rates of overweight and obesity compared to their counterparts in rural areas, as well as those in Mexico City and the central regions (38). Street connectivity, residential density, access to green spaces, public transportation, sidewalks, fast-food restaurants, and fresh markets are factors within urban areas that may have a significant role in the onset of childhood obesity (39, 40).

A recent meta-analysis comprising 457 studies revealed that most built environmental factors were inversely associated with childhood obesity. Specifically, access to green spaces was associated with increased PA and reduced screen time (38). Furthermore, access to food outlets, excluding convenience stores and fast food, was also correlated with healthier dietary behaviors. In contrast, greater proximity to fast-food restaurants was linked to higher consumption of ultra-processed foods, contributing to the creation of an obesogenic environment (41).

The built environment, such as transportation infrastructure and recreational facilities, may influence individual behaviors, potentially leading to reduced PA and increased obesity rates among children and adolescents (40). According to recent results, green and blue spaces play a significant role in promoting PA and influencing eating behaviors among children under 18 years old from various countries, including New Zealand, the UK, the USA, the Netherlands, Canada, Turkey, and Germany. Green spaces encompass parks, sports fields, playgrounds, nature reserves, and open picnic areas, while blue spaces include lakes, rivers, canals, and waterfronts. Green and Blue Spaces (GABS) provide safe environments for children to socialize and engage in lengthy and enjoyable PA. Additionally, the creation of school and home gardens has a positive impact on children's attitudes toward eating vegetables, thus promoting healthier dietary habits (39).

### 2.4 Climate change

The decline in the nutritional quality of food due to rising CO_2_ levels and increasing global temperatures has significant implications for human health, particularly through the exacerbation of hidden hunger. The reduction of essential micronutrients such as iron, zinc, and protein in staple crops like wheat, rice, and maize (42, 43) contributes to widespread deficiencies, potentially affecting hundreds of millions of people by 2050 (44–46). Hidden hunger, a condition in which individuals consume sufficient calories but lack essential nutrients, is expected to increase by 10% due to climate-driven decreases in nutrient bioavailability (47). Additionally, extreme weather events and high ambient temperatures further compromise agricultural productivity, reducing protein content and overall crop yields (48–50).

These nutritional deficits are compounded by environmental contaminants, as climate change enhances the bioaccumulation of heavy metals like arsenic in food crops, particularly rice, leading to toxic effects on the gut microbiota (51, 52). Warmer waters similarly diminish omega-3 fatty acid concentrations in marine food webs, weakening their beneficial effects on gut health and immune function (53). Beyond direct dietary impacts, prolonged exposure to heat stress increases gut permeability, disrupts microbial balance, and promotes systemic inflammation, heightening susceptibility to gastrointestinal disorders such as inflammatory bowel disease (54–56). Furthermore, extreme temperatures exacerbate malnutrition in vulnerable populations, particularly children in low- and middle-income countries, where dysbiotic gut microbiota may persist despite nutritional interventions (57).

Breastfeeding patterns may be altered under high heat stress, influencing early-life gut microbiome development and potentially reinforcing health disparities (58–60). The interplay of these factors underscores the urgent need for climate-resilient agricultural strategies, biofortification efforts, and microbiota-targeted interventions to mitigate the long-term health consequences of climate change on human nutrition and gut health.

## 3 Specific external exposome and childhood obesity

During the “first 1,000 days” feeding practices, maternal substance consumption, and infant lifestyle factors are key components of the specific external exposome and may affect the risk of childhood obesity (61–63).

### 3.1 Infant feeding practices

Concerning the role of breastfeeding, several studies support the evidence that exclusively breastfed infants are at lower risk of accumulating excessive fat mass and experiencing overweight and obesity, in comparison with formula-fed infants (64, 65).

This protective effect can be attributed to the unique composition of human breast milk (HBM), which contains metabolic hormones, bioactive molecules, and essential nutrients (66, 67). For example, leptin, ghrelin, growth factors, and hormones play a crucial role in regulating children's food intake and energy balance, controlling appetite, glucose, and lipid levels (68).

Human milk oligosaccharides (HMOs), which are highly concentrated in HBM, promote the growth of beneficial gut bacteria and may influence infant development, including weight gain and body composition (69). A recent systematic review conducted by Zheng et al. (68) summarized findings from 27 studies to assess the association between breastfeeding and BMI trajectory changes over time between childhood and adulthood. Findings revealed that breastfeeding, whether exclusive or combined with formula feeding, is associated with lower BMI trajectories compared to exclusively formula-fed infants (68). Moreover, the WHO conducted a study on 16 European countries to examine the relationship between different feeding practices, and children's body weight. The results showed a clear protective effect of exclusively breastfeeding, reducing the risk of obesity by 25%. On the other hand, an increased risk of 22% was observed among exclusively formula-fed infants (70, 71). The study also highlighted the influence of breastfeeding duration on the risk of obesity, showing that children breastfed for <6 months had a 12% increased risk (70, 71). However, a systematic review published in 2023 revealed conflicting evidence regarding the composition of HBM and the subsequent risk of obesity (72). The review investigated the role of hormones present in HBM, such as leptin, adiponectin, and insulin, in relation to the risk of later obesity, finding heterogeneous results. Additionally, the association between the macronutrient composition of HBM and the risk of subsequent obesity or body composition was examined, with only one study identifying that a higher fat percentage in HBM was associated with lower adiposity at 12 months, while a higher carbohydrate percentage was linked to increased adiposity at the same age, independent of other factors (72).

These findings emphasize the need for a better understanding of the mechanisms supporting the protective effects of breastfeeding against later obesity and highlight the necessity for further research in this area (72).

#### 3.1.1 Human breast milk as a vehicle of EDCs and nicotine

Although HBM represents the primary and healthiest nutritional source, it may also serve as a significant vehicle for environmental pollutants and various harmful substances, such as EDCs, and nicotine (73, 74). Vulnerable groups including fetuses, infants, and children, may face greater susceptibility to environmental chemicals due to differences in toxicokinetics, resulting in an elevated risk of childhood diseases (75). In 2022, Iribarne-Durán et al. published the first results about the concentrations of some EDCs in HBM (73), suggesting that the entero-mammary circulation facilitates the transfer of these chemicals, as certain EDCs can cross the gut barrier, enter the bloodstream, and reach the mammary glands, subsequently appearing in HBM (76) and potentially exposing the infant to harmful effects (77). A recent study conducted by Vacca et al. investigated the association between maternal urinary concentration of EDCs and the gut microbiota composition of 20 breastfed infants, at four time points. The authors identified that maternal EDCs exposure impacts the infant's gut microbiota and potentially influences the risk of metabolic and inflammatory diseases including obesity (78).

Early-life exposure to smoking was also found to be associated with childhood obesity (79, 80). Specifically, nicotine quickly transfers into HBM, potentially harming an infant's development and health (79, 80).

Exposure to tobacco smoke during pregnancy and lactation has been linked to changes in the macronutrient composition of HBM. This may be due to the accumulation of toxic substances in the adipose tissue of the mammary glands, potentially disrupting lactogenesis and lipid synthesis (81).

In this regard, a systematic review conducted by Macchi et al. revealed that breastfed children of smoking mothers have reduced lean body mass and an increased risk of developing obesity within their first year of life (81). Moreover, tobacco exposure leads to a significant rise in thiobarbituric acid reactive substances (TBARS) levels, a marker of lipid peroxidation, and a reduction in trolox equivalent antioxidant capacity (TEAC), a measurement of total antioxidant capacity, within both colostrum and mature milk. In response to the increased reactive oxygen species (ROS) induced by tobacco smoke, there is a corresponding upregulation in the activity of antioxidant enzymes (82). These oxidative changes may contribute to the alterations observed in breast milk composition and potentially impact infant health (82). Several studies have also shown that nicotine not only passes through HBM but also crosses the placenta (83).

As a result, exposure to maternal or paternal smoking during pregnancy has been identified as a risk factor for the development of early overweight (83). A meta-analysis of 229,000 births showed that children from mothers who smoked during pregnancy had an increased risk of developing overweight [OR 1.42 (1.35–1.48), *P*-value <0.001] compared to children from no-smoking mothers. Noteworthy, paternal tobacco consumption was also assessed, revealing an association with a higher risk of childhood overweight, independently of maternal smoking habits (83). A study conducted by Srivastava et al. (79) revealed high rates of obesity in children exposed to parental smoking with the association being stronger with maternal smoking than with paternal smoking (79). This was confirmed in another study conducted by Cummings et al. (80), where the authors identified that a family history of nicotine use, and alcohol consumption was accompanied by an increased reward-driven eating in their children. Such behaviors may later lead to overweight and obesity, since children may eat more for pleasure rather than for satiety (80).

#### 3.1.2 Complementary feeding

The transition from exclusive breast milk feeding to solid foods also plays a significant role in children's weight change, based on the timing of its introduction as well as its composition (71, 84, 85). The complementary feeding period is essential for providing children with safe and nutrient-dense foods and preventing overweight and obesity, as well as influencing their future dietary preferences (85). Early introduction of complementary feeding, before 6 months of age, was shown to be associated with an increased risk of obesity (84). According to WHO and UNICEF recommendations, exclusive breastfeeding for the first 6 months of life must be followed by continued frequent or on-demand breastfeeding, combined with complementary feeding up to 2 years of age (71, 85). Caregivers play an essential role in protecting their infants and toddlers from the risk of excessive weight gain by providing them with an age-appropriate complementary diet, that is rich in nutritive value and includes a variety of foods from all food groups. This diet should be low in saturated fats, and trans fats and totally free from added sugars and salt (85). In this regard, a systematic review and meta-analysis was conducted by Rousham et al. to assess the impact of consuming unhealthy foods and beverages on the risk of overweight and obesity in children aged 10.9 years or below, in comparison with no or lower consumption levels (86). This review found a positive association between the consumption of sugar-sweetened beverages and both BMI level and body fat percentage, whereas artificially sweetened beverages or 100% fruit juice, had a low or no impact on BMI levels. On the other hand, unhealthy foods including ultra-processed items were found to increase BMI levels and obesity risk (86).

### 3.2 Children's lifestyle

Children's lifestyle is a significant component of early-life exposome that can impact body weight (87). Regarding daily screen time, the systematic review and meta-analysis conducted by Fang et al. (63) revealed that screen time ≥2 h per day was more strongly associated with an increased risk of overweight/obesity compared to screen time of <2 h per day. Additionally, the analysis showed that specific types of screen time, such as TV viewing and computer use, were more strongly linked to overweight and obesity than total screen time. Many existing studies have focused on the impact of a single type of screen time, which may not fully capture the overall effects of screen time on childhood obesity. Therefore, it is important to differentiate between the effects of different types of screen time when assessing their influence on childhood obesity. As recommended by WHO, children under 5 years old should limit sedentary behaviors, including screen time and prolonged periods spent in their strollers or chairs, ensure adequate sleep duration, and engage in active play to achieve healthy growth from the beginning of their lives and prevent childhood obesity and its related consequences (87). PA level must be incorporated in the child's daily routine, whether at home, in the nursery, or at school (88). Together with the quality of complementary feeding, PA can maintain energy balance and reduce the risk of overweight and obesity (88). It could be influenced by different factors including weather conditions (hot or cold degrees, wind speed, and precipitation (89, 90). A recent systematic review conducted by Jia et al. (89) showed that high and low temperatures were significantly linked to reduced daily PA levels among children (89). Another longitudinal prospective cohort study conducted on 372 children aged 3 years and followed for 5 years, showed that precipitation, wind speed, higher heating and cooling degrees than the average temperature, were associated with a decreased PA level (90). Apart from PA, sleep duration was also found to have a link with the risk of overweight and obesity. As demonstrated in the systematic review conducted by Morrissey et al. (91) a strong negative association was detected between insufficient sleep duration and increased weight status in primary school-aged children (91).

### 3.3 Socioeconomic status

Another important determinant of the specific external exposome is represented by the SES, recognized as a significant determinant of numerous adverse health outcomes, including obesity as obesity prevalence is inextricably related to the degree of relative social inequality (92). Although children do not have their own SES, recognized contextual factors include the parental sociodemographic characteristics (e.g., age and sex, race or ethnicity, SES) as a proxy for a child's SES level (93). Recent research categorized contextual factors to the child, parents, or family (e.g., sex, age, race, or ethnicity) and various SES metrics (e.g., annual family income, education level and/or employment of parents, health insurance coverage, and eligibility for free school lunch program) to investigate whether these factors serve as moderators in the relationship between parental stress and childhood obesity (93). Results revealed that parenting role stress may be associated with unhealthy practices such as children's unhealthy food intake, including consumption of fast foods, emotional overeating, screen time, and low PA levels (93). In this regard, recent studies have shown that dietary practices, sleep time, and level of PA in children aged 6-12 years living in the Pacific Region play a significant role in the development of overweight and obesity. Moreover, SES and food availability, parenting practices, and education level contribute to children's weight status (37, 94). Concerning the educational level of the family, while current literature does not directly address its relationship with childhood obesity, findings suggest that socioeconomic factors and parenting practices, which may be influenced by educational attainment, may play a significant role (94, 95). Bertrand and colleagues identified that the education level of caregivers was a key determinant of children's weight status, with higher caregiver education associated with a greater likelihood of childhood obesity in certain contexts. However, these findings are in contrast with other data, where an inverse association was registered between fathers' educational attainment and daughters' adiposity (94). Recently, Alruwaii conducted a systematic review examining the impact of both parents' educational levels on childhood obesity and overweight in Middle Eastern and North African countries, emphasizing the interaction between SES and metabolic health outcomes (95).

Although family-based SES indicators include several parameters, such as parental education level, occupation, living conditions, size of family, family income, and type of medical insurance, SES emerged as one of the most investigated parental factors. McGillivray and colleagues have reported a positive association between SES and BMI in children with intellectual or developmental disability; however, other research did not find a significant statistical association (96).

SES may also influence dietary habits among children. Notably, findings by Avery and colleagues examined the associations between TV viewing while eating and children's diet quality (97). Four studies in their systematic review identified an association between low SES and increased likelihood of eating while watching TV (*p* ≤ 0.01), highlighting the need for educational programs targeting parents, especially those with low-socioeconomic backgrounds (97).

In this context, nutritional education intervention may play a significant role in the prevention and treatment of obesity, mostly during early life. Recently, Spiga and colleagues collected data to explore the effectiveness of educational interventions focused on dietary and/or PA aimed at preventing childhood obesity, depending on factors related to health disparities, such as SES (98). Exploratory analyses of 55 studies targeting low-SES populations found no evidence suggesting that obesity prevention interventions are less effective in children from lower socioeconomic backgrounds (98).

Current literature also explores the role of the neighborhood environment on childhood obesity onset. A systematic review examined the neighborhood environment and obesity risk among urban, low SES Black and Hispanic children (99). Among the 24 included studies, 16 reported an association between neighborhood SES and BMI for the overall study population. These primarily investigated the relationship between neighborhood SES and BMI as measured by neighborhood income or a composite SES measure. These composite indicators integrate various SES-related factors, such as educational attainment, employment status, household income, and financial wellbeing, to generate a single score (100). While four studies found no association between composite SES and BMI, one study identified an inverse relationship. Similarly, three studies showed no association between neighborhood income and BMI, while five studies reported an inverse relationship (99).

## 4 Internal exposome and childhood obesity

The internal exposome comprises a plethora of biological responses occurring within the human body due to exposure to external stimuli (101). Metabolic processes that begin in childhood can increase the risk of obesity and other long-term health complications (102).

### 4.1 Exposure to chemical compounds

Considering exposure factors related to the general external exposome, the activation of the PPARs pathway has been implicated in the metabolic effects of phthalates and BPA exposures, which can enhance the risk of obesity by interfering with several pathways. In particular, these chemicals may (i) disrupt adipogenesis by inducing ROS species production, which can interfere with the normal differentiation of adipocytes; (ii) increase the number and size of adipocytes by regulating genes involved in adipogenesis; (iii) alter epigenetic pathways during development, which increases susceptibility to obesity; (iv) disrupt neuroendocrine signals involved in appetite and satiety pathways; (v) foster a proinflammatory environment in adipose tissue, leading to chronic low-grade inflammation; (vi) disrupting the gut microbiome and immune system balance; and (vii) impair the function of thermogenic adipose tissue (103–105). Recent evidence found that phthalates and BPA can pass through HBM, potentially affecting infant health (106, 107). In particular, high molecular weight phthalates and di(2-ethylhexyl) phthalate (DEHP) metabolites have been also linked to increased visceral adipose tissue (VAT) mass and higher Android-to-Gynoid (A/G) ratio in adolescents (108).

A significant association has been observed, with 5-fold increases in phthalate metabolites correlating to 21.7% and 18.0% greater VAT mass, respectively (106). Additionally, recent studies have investigated the role of per- and polyfluoroalkyl substances (PFAS) in childhood obesity. A systematic review and meta-analysis of 13 studies found no strong evidence of a direct association; however, an inverse relationship was suggested between postnatal PFAS exposure and BMI *z*-score. The limited number of studies available on this topic warrants the need for further investigation (17). In summary, exposure to environmental factors during both the prenatal period and early life can disrupt key metabolic pathways, affecting adipogenesis, lipid and glucose metabolism, gut microbiota homeostasis, and growth trajectories, with long-term implications for a child's health, including a higher risk of developing obesity. The interaction between genetic susceptibility, environmental exposures, and early-life exposure factors, such as breastfeeding, offers new insights into the complex mechanisms driving obesity risk.

### 4.2 Infant feeding practices

Referring to the specific external exposome, the type of infant feeding is an important factor influencing the risk of childhood obesity. Exclusive breastfeeding until the age of 2 is strongly recommended as a protective factor for the risk of obesity in children (109, 110). Research indicates that this practice may affect obesity-related gene expression, including fat mass and obesity-associated gene (FTO), Nuclear Respiratory Factor 1 gene (NRF1), and Leptin Receptor gene (LEPR), through epigenetic processes such as DNA methylation and regulation of CpG island loci (111). Studies have demonstrated that breastfeeding delays the onset of adiposity peaks and rebounds, helping to prevent excessive weight gain, particularly in children with a genetic predisposition (112, 113).

The FTO gene is essential for cell proliferation and differentiation through the PI3K/Akt signaling pathway; it also interacts with AMP-activated protein kinase (AMPK) and the PI3K/AKT/mTOR pathways, which are key regulators of energy metabolism (113). Recent research has emphasized the contribution of the FTO gene, especially its polymorphism, in promoting increased BMI and adiposity in children (114, 115). A study conducted by Wu and colleagues revealed that exclusive breastfeeding up to 5 months significantly reduces the risk of obesity in children carrying the FTO rs9939609 risk allele. The study further indicated that breastfeeding postpones the age at which peak fat mass and fat accumulation occur. Specifically, breastfed children experienced a delay of 2–3 months in reaching their peak fat compared to non-breastfed children, with girls showing a delay of up to 6 months. At age 15, the adolescents exhibited a predicted BMI reduction of 0.56 kg/m^2^ for boys [CI 95%:0.11–1.01; *P* = 0.003] and 1.14 kg/m^2^ for girls [CI 95%:0.67–1.62; *P* < 0.0001] (116). These findings highlighted the role of exclusive breastfeeding in mitigating up to 39–70% of genetic obesity risk, particularly in children characterized by high genetic risk scores (116). Also, Verier et al. highlighted the significant interaction between breastfeeding and polymorphisms of the peroxisome proliferator-activated receptor γ (PPAR-γ) gene concerning childhood obesity. Prolonged breastfeeding in children with the high-risk variant Pro12Ala phenotype led to reduced BMI, waist circumference, and skinfold thickness compared to formula-fed children (117). However, for children with the non-high-risk Pro12Pro phenotype, breastfeeding duration had no significant effect on obesity-related indicators (117). The human PPAR genes are involved in regulating lipid and glucose metabolism, lipid storage, and insulin sensitization. Given the gene's role in macronutrient metabolism, the PPAR signaling pathway has become a focal point in obesity research, particularly in elucidating the interactions between environmental exposure and gene expression (118).

#### 4.2.1 Complementary feeding

The introduction of solid foods represents a significant milestone in the development of various infant physiological systems, including the gastrointestinal tract, gut microbiota, and immune system (119). Evidence suggests that initiating solid food consumption at approximately 5–6 months of age may correlate with a reduced risk of greater BMI. However, systematic reviews have emphasized the need for additional prospective studies to better assess the differences between the introduction of solid food and exclusively breastfed, formula-fed, or mixed-fed infants (120, 121). Additionally, the dietary pattern adopted during the introduction of solid foods is particularly relevant, as it can influence behavioral outcomes and molecular pathways that may affect the risk of obesity (122). In fact, in their Randomized Controlled Trial (RCT) including healthy, full-term formula-fed infants, Tang et al. (123) demonstrated that the *z*-score for length-for-age significantly increased in the group of children with higher consumption of meat (+0.33 ± 0.09; *P* = 0.001 over time), whereas it decreased in the dairy group (−0.30 ± 0.10; *P* = 0.0002 over time). Moreover, the *z*-score for weight-for-length increased significantly in the dairy group (0.76 ± 0.21; *P* = 0.000002 over time) compared to the meat group (0.30 ± 0.17; *P* = 0.55 over time) (123). Although the WHO guidelines recommend daily or frequent consumption of animal-source foods such as meat, poultry, fish, or eggs due to their high nutrient density, providing easily digestible proteins, several studies and systematic reviews have shown that higher protein intake before the age of 2 is related to accelerated growth trajectories and increased risk of higher BMI later in childhood (124, 125). According to the early protein hypothesis, high protein intake during lactation and complementary feeding is thought to stimulate insulin and insulin-like growth factor (IGF) secretion, which can promote fat accumulation by enhancing adipogenesis and adipocyte differentiation (126, 127). However, other research has found no significant differences in IGF-I levels among infants consuming varying amounts or sources of protein, indicating that other mechanisms may occur (123). The development of gut microbiota is a complex process, beginning at birth and influencing long-term health. A first factor implicated in the development of the intestinal microbiota of a newborn is the mode of delivery, i.e., natural birth or c-section, followed by the immediate feeding method, i.e., breastfeeding or formula feeding. This sequence of events and future feeding habits is crucial for the long-term development of the intestinal microbiota. The infant gut is initially colonized by facultative anaerobes, such as Staphylococcus, Streptococcus, Enterobacteriaceae, and Lactobacillus, which create an environment suitable for obligate anaerobes like *Bifidobacterium, Clostridium*, and *Bacteroides* to thrive (128, 129). Maternal milk, with its nutritional and bioactive components, fosters the optimal microbial growth in the infant gut, influencing both the microbiota's composition and immune system development.

Therefore, the introduction of semi-solid and solid food significantly alters the composition of the gut microbiota, contributing to its maturation and diversification (130). During this transition, the abundance of milk-related bacteria, such as Bifidobacterium and Enterobacteriaceae, decreases, while bacteria such as *Bacteroides* and *Firmicutes*, which preferentially digest fibers and complex carbohydrates, increase (131). Comparisons of different dietary patterns revealed that children following a Mediterranean Diet (MD) exhibited greater intestinal microbial diversity and a higher abundance of beneficial taxa, such as *Coriobacteriaceae*, which can metabolize polyphenols, particularly abundant in the MD (130). Recent research has increasingly highlighted the role of the gut microbiota as a key mediator in the development of obesity, particularly during early life (132). Bacteria from the *Firmicutes* and *Bacteroidetes* phyla are closely linked to the regulation of energy metabolism. A dysbiotic microbiota can lead to reduced production of short-chain fatty acids (SCFAs), which may promote systemic inflammation and, consequently, insulin resistance and visceral fat accumulation (130, 133).

A fiber-based diet favors SCFA-producing bacteria implicated in obesity and in the regulation of intestinal endocrine signals, influencing glucose and lipid metabolism. Furthermore, SCFAs are implicated in the regulation of oxidative metabolism and insulin sensitivity in the liver and adipose tissue, thus managing to improve obesity, determining the reduction of metabolic endotoxemia and inflammation (134, 135). SCFAs play a crucial role in preserving the integrity of the barrier, and *Bacteroides thetaiotaomicron*, through the production of acetate and propionate, regulates the production of mucin; therefore, the balance of the intestinal mucosa (136).

### 4.3 Maternal smoking habits

Moreover, the maternal smoking habit during pregnancy can affect the risk of excessive weight gain during early life. Peng and colleagues demonstrated that infants exposed to maternal smoking during gestation had higher BMI *z*-scores at 3 years of age than those who were not exposed (Model 3: β = 0.28, CI: 95%; 0.06–0.49). They were significantly more likely to be affected by obesity at 3 years of age (Model 3: OR 1.78, CI: 95%; 1.11–2.86) (134). Additionally, gut microbiota mediated the effect of smoking habits during pregnancy on the higher risk of obesity in offspring. Particularly, the Firmicutes group accounted for the largest portions (23.3–24.6%) of the total effects on BMI *z*-scores at 1 year and 12.4–15.2% at 3 years of age of the children (137). The main results are summarized in Table 1.

**Table 1:** Summary of the main results of the studies included.

**Infant exposome domains**	**Exposure factors**	**Study design**	**References**	**Main results of the studies**
General external exposome	Air quality	Analysis from a longitudinal cohort study (29)	Margetaki et al. (29)	•Exposure to PMs *in utero* was not associated with measures of adiposity at 4 or 6 y (29); •Higher exposure to PM_10_ during pregnancy, combined with maternal consumption of <5 servings of FV/day was associated with increased BMI (beta 0.41 kg/m^2^, 95% CI: −0.06, 0.88, p-interaction = 0.037) and increased WC (beta 0.83 cm, 95% CI: −0.38, 2.05, p-interaction = 0.043) in children at 6 years (29); •Higher exposure to PM_2.5_ during pregnancy, combined with maternal consumption of <5 servings of FV/day were associated with increased fat mass (beta 0.5 kg, 95% CI: 0.0, 0.99, p-interaction = 0.039) and percentage of body fat (beta 1.06%, 95% CI: −0.06, 2.17, p-interaction = 0.035) in children at 6 years (29).
		Systematic review and meta-analysis (25)	Huang et al. (25)	•Higher exposure to air pollutants was significantly associated with higher obesity risk [OR = 1.12 (95% CI: 1.06-1.18) for PM_10_, OR = 1.28 (95% CI: 1.13-1.45) for PM_2.5_, OR = 1.41 (95% CI: 1.30-1.53) for PM_1_, and OR = 1.11 (95% CI: 1.06-1.18) for NO_2_] (25); •Each 10 μg/m3 increment in pollutant concentration was associated with BMI increases of +0.08 kg/m^2^ (95% CI: 0.03-0.12) for PM_10_, +0.11 kg/m^2^ (95% CI: 0.05-0.17) for PM_2.5_, and +0.03 kg/m^2^ (95% CI: 0.01-0.04) for NO_2_ (25).
		Systematic review and meta-analysis (17)	Frangione et al. (17)	•No evidence of a positive association was found between prenatal PFAS exposure and pediatric obesity (17); •Prenatal PFAS exposure was not statistically associated with BMI or WC, whereas postnatal exposure showed inverse associations (17).
		Analysis from a longitudinal cohort study (26)	Shao et al. (26)	•Prenatal exposure to PM_2.5_, PM_10_, SO_2_, and O_3_ was associated with reduced fetal biometry, specifically at GW24 (SO_2_ strongest effect: +10 μg/m3 femur length −2.20 mm, ≈−5.3%). Effects persisted but were attenuated at GW36 (26); •No differences in birth weight were registered, indicating rapid catch-up growth in the 3rd trimester (26).
	EDCs	Analysis from a longitudinal cohort study (108)	Webb et al. (108)	•5-fold increase in HMW (%BF: +2.86 units, 95% CI: 0.69–5.03; % of VAT+21.7%, 95% CI: 10.5–33.9) and DEHP (% of BF: +2.69 units, 95% CI: 0.66–4.72; VAT:) exposure was associated with increased %BF and VAT (+18.0%, 95% CI: 7.72–29.2) in adolescent males (108) •5-fold increase in LMW exposure was associated with increased %BF (+2.01 units, 95% CI: 0.05–3.98), VAT (+9.38%, 95% CI: 0.01–19.6), VAT (+9.38%, 95% CI: 0.01–19.6) and A/G ratio (+0.03, 95% CI: 0.00–0.07) in adolescent males (105); •No significant associations in adolescent females (108).
		*In vitro* study (104)	Longo et al. (104)	•BPA induces hypomethylation of the Pparγ promoter and transient gene activation (*p* < 0.05) (104); •BPA stimulates transient lipid accumulation and inflammation, both reversible (*p* < 0.01; proinflammatory cytokines Il6, Ifnγ, Tnfα, Mcp1, Il1β increased during exposure, normalized after BPA removal) (104);
		Systematic review (105)	Naomi et al. (105)	•BPA exposure was associated with lower abundance of *Bifidobacterium* spp. (4.2% vs. 7.8%, *p* < 0.01) and *Clostridium* Cluster XIVa (6.5% vs. 10.3%, *p* < 0.05), lower SCFAs production (1.8 vs. 3.5 μmol/g, *p* < 0.01), and a higher abundance of *Proteobacteria* (9.2% vs. 3.7%, *p* < 0.01) (105); •Sex-dependent differences were observed: males had higher *Bacteroides, Mollicutes, Prevotellaceae*, and *Akkermansia*, while females had higher *Lachnobacterium* and *Prevotella (105)*.
	Urbanization	Systematic review and meta-analysis (36);	Johnson et al. (36)	•Across 10 studies (five in meta-analysis, *n* = 74,168 children, 2–19 years), rural residence was consistently associated with higher obesity prevalence. Pooled data showed 26% greater odds of obesity in rural vs. urban children (OR = 1.26; 95% CI: 1.21–1.32) (36);
	Nbs	Cross-sectional analysis from a longitudinal cohort study (33)	Heo et al. (33)	•Prenatal greenspace exposure (EVI, park number) was linked to a reduced risk of preterm birth (Q2 OR = 0.65; Q3 OR = 0.51; Q4 OR = 0.56 vs. Q1) (33). •No significant associations were found with the terms low birthweight, birthweight, or estimated fetal weight (33).
		Cross-sectional analysis from 11 cohort studies (34)	Torres Toda et al. (34)	•An IQR increase in residential surrounding greenspace (100 m, 300 m, 500 m) was associated with lower odds of SGA (OR = 0.87, 95% CI: 0.83–0.92; OR = 0.87, 95% CI: 0.82–0.91; OR = 0.86, 95% CI: 0.81–0.90) (34); •Greater residential distance to greenspace increased SGA risk (OR = 1.07, 95% CI: 1.02–1.12). Associations for accessibility to GABS exposure were close to null (34).
	Climate	Meta-analysis (42)	Myers et al. (42)	•CO_2_ (546–586 ppm) significantly decreased nutrient concentrations: wheat grains showed −9.3% zinc (95% CI: −12.7 to −5.9), −5.1% iron (95% CI: −6.5 to −3.7), and −6.3% protein (95% CI: −7.5 to −5.2), while rice showed −7.8% protein (95% CI: −8.9 to −6.8) (42); •Reductions in zinc and iron were observed across other C3 legumes and grasses, whereas C4 crops were minimally affected (42).
		Analysis from a longitudinal cohort study (58)	Part et al. (58)	•Each +1 °C increase in daily mean temperature was associated with −2.3 min/day BF (95% CI: −4.6 to 0.04) and +0.6 min/day childcare (95% CI: 0.06–1.2). During the hottest vs. coolest periods, women breastfed ~25 min/day less. Odds of exclusive BF in very young infants (0–3 months) decreased with higher temperature (OR = 0.88; 95% CI: 0.75–1.02) (58). •No associations were found in exclusively breastfed infants at 3–6 months or for supplementary feeding at 6–12 months (58).
Specific external exposome	Breastfeeding	Systematic review (110)	Kumari et al. (110)	•Protective effect of prolonged BF on excess child body weight (110).
		Analysis from a longitudinal cohort study (111)	Lin et al (111)	•Lower risk of EAR in children breastfed for > 4 months (adjusted RR = 0.80, 95% CI: 0.73-0.87, *p* < 0.001) (111).
		Analysis from a longitudinal cohort study (112)	Wu et al. (112)	•Delayed AR in girls: 5 months EBF associated with +0.64 years (GRS 2.5), +0.53 years (GRS 5.0), +0.44 years (GRS 7.5); *p* < 0.05 (112); •Delayed AP: 5 months EBF associated with +0.21 years (GRS 5.0), +0.25 years (GRS 7.5) in boys and +0.14 years (GRS 2.5), +0.24 years (GRS 7.5) in girls; *p* < 0.05 (112); •BMI reduction at 18 y with 5 months EBF: boys −0.81 to −1.14 kg/m^2^ and girls −0.86 to −1.53 kg/m^2^ (depending on GRS); *p* < 0.05 (112); •Non-exclusive BF associated with lower BMI reduction compared to EBF: −0.31 to −0.37 kg/m^2^ in boys, −0.34 to −0.54 kg/m^2^ in girls (*p* < 0.05) (112); •Shorter EBF (3 months) was associated with smaller effects on delaying AP/AR and reducing BMI (boys −0.49 to −0.68 kg/m^2^, girls −0.52 to −0.92 kg/m^2^; depending on GRS; *p* < 0.05) (112).
		Analysis from a longitudinal cohort study (114)	Kanders et al. (114)	•BF 7–12 months: lower risk of overweight at W1 (114); •BF >12 months in FTO rs9939609 TA children was associated with reduced risk of overweight at W1 (OR = 0.41, 95% CI: 0.19–0.88) (114); •No significant effect of BF or FTO variants on BMI/overweight at W2 or W3 (114).
		Analysis from a cross-sectional study (117)	Verier et al. (117)	•Non–breastfed children (*n* = 173), Ala12 carriers vs. Pro12Pro registered higher BMI: +1.88 kg/m^2^ (adjusted *P* = 0.007), WC: +3.8 cm (adjusted *P* = 0.03) (117); •Breastfed children: no significant difference in BMI, WC, or skinfolds between Ala12 carriers and Pro12Pro (117); •Protective effect of BF mitigates genetic predisposition to higher adiposity in Ala12 carriers, even for short-duration BF (117).
	Breast milk (as a vehicle of substances) *EDCs*	Analysis from a longitudinal cohort study (78)	Vacca M. et al. (78)	•In breastfed infants (stratified by maternal urinary BPA; >0.96 mg/g creatinine = high exposure), differential gut colonization was observed at 12 months: *Ruminococcus torques* group was significantly higher in low-exposed infants (*p* < 0.05), whereas *Erysipelatoclostridium* and *Bifidobacterium breve* were enriched in high-exposed infants (78); •Retrospective β-diversity analysis (from birth to 12 months) confirmed compositional disparities between exposure groups (78); •Stratification by phthalate exposure showed no significant differences between groups (78).
	*Nicotine*	Cross-sectional analysis from a cohort study (82)	Napierala et al. (82)	•Smoking during lactation significantly increased the TBARS and TEAC (measure of total antioxidant potential) in colostrum and mature milk (*p* < 0.05) (82); •The activity of antioxidant enzymes, including superoxide dismutase, glutathione S-transferase, glutathione peroxidase, and catalase, was significantly elevated (*p* < 0.05) (82).
		Systematic review (81)	Macchi et al. (81)	•Maternal smoking during lactation was consistently associated with altered HBM composition. Specifically, smoking is correlated with a reduction in total lipid and PUFA levels and a higher concentration of MUFA (81).
		Analysis from a longitudinal cohort study (79)	Srivastava et al. (79)	•Maternal smoking increased obesity probability by +2.8%, paternal smoking by +2.1%, and exposure to both parents by +2.0%. Age-stratified analyses confirmed a higher risk in (4–11 years: +2.3%) children (79)
	Complementary feeding	Analysis from 2 cohort studies (119)	Homann et al. (119)	•Higher daily dietary diversity modulated the gut microbiota homeostasis and promoted taxa as *Bifidobacterium* (β = 0.28, *p* < 0.01), while reducing genera like *Veillonella* (β = −0.22, *p* < 0.05) (119).
**Internal exposome**	**Biological responses to external exposure factors**			
	ROS production (82) Adipogenesis genes expression (107) Epigenetic pathways *via* DNA methylation and CpG island regulation (104, 107) Chronic low-grade inflammation in adipose tissue (104) Gut microbiota homeostasis (116, 125) SCFAs production (105) Obesity-related genes (FTO, NRF1, LEPR) and PPAR signaling (104, 107, 111)			

Presentation of the findings referred to the exposome domains and biological responses.

PM, particulate matter; FV, fruits and vegetables; BMI, body mass index; WC, waist circumference; PFAS, per- and polyfluoroalkyl substances; GW, gestational age; EDCs, endocrine disrupting chemicals; HMW, high molecular weight; DEHP, di(2-ethylhexyl) phthalate; %BF, percentage body fat; VAT, visceral adipose tissue LMW, low molecular weight; A/G ratio, Android-to-Gynoid fat ratio; BPA, bisphenol A; PPAR, peroxisome proliferator-activated receptor; SCFAs, short-chain fatty acids; Nbs, nature-based solution; EVI, enhanced vegetation index; SGA, small for gestational age; BF, breastfeeding; EAR, early adiposity rebound; AR, adiposity rebound; EBF, exclusive breastfeeding; AP, adiposity peak; FTO, fat mass and obesity-associated gene; TBARS, thiobarbituric acid reactive substances; TEAC, trolox equivalent antioxidant capacity; HBM, human breast milk; PUFA, polyunsaturated fatty acid; MUFA, monounsaturated fatty acid; ROS, reactive oxygen species; NRF1, nuclear respiratory factor 1 gene; LEPR, Leptin Receptor gene.

## 5 Discussion

Childhood obesity has become one of the most critical global health challenges (5). Particular attention should be directed to preventable factors during the pivotal period of the “first 1,000 days” (6). This narrative review provides a novel perspective by exploring various exposure factors within the general external exposome during the “first 1,000 days” of life (chemical compounds, air pollution, urbanization), focusing on their potential role in the development of childhood obesity (17, 23, 36).

In addition, the review also explores specific external exposome factors (infant feeding practices, children's lifestyle, SES) to which the mother, and more broadly the family, are exposed, recognizing the complex interplay of intergenerational and familial dynamics that may affect the child's development and future health (71, 93). Furthermore, exposure to environmental factors during both the prenatal period and early childhood may alter crucial metabolic processes, threatening the internal exposome homeostasis and increasing the risk of developing obesity and other metabolic disorders (102).

**Figure 1:**
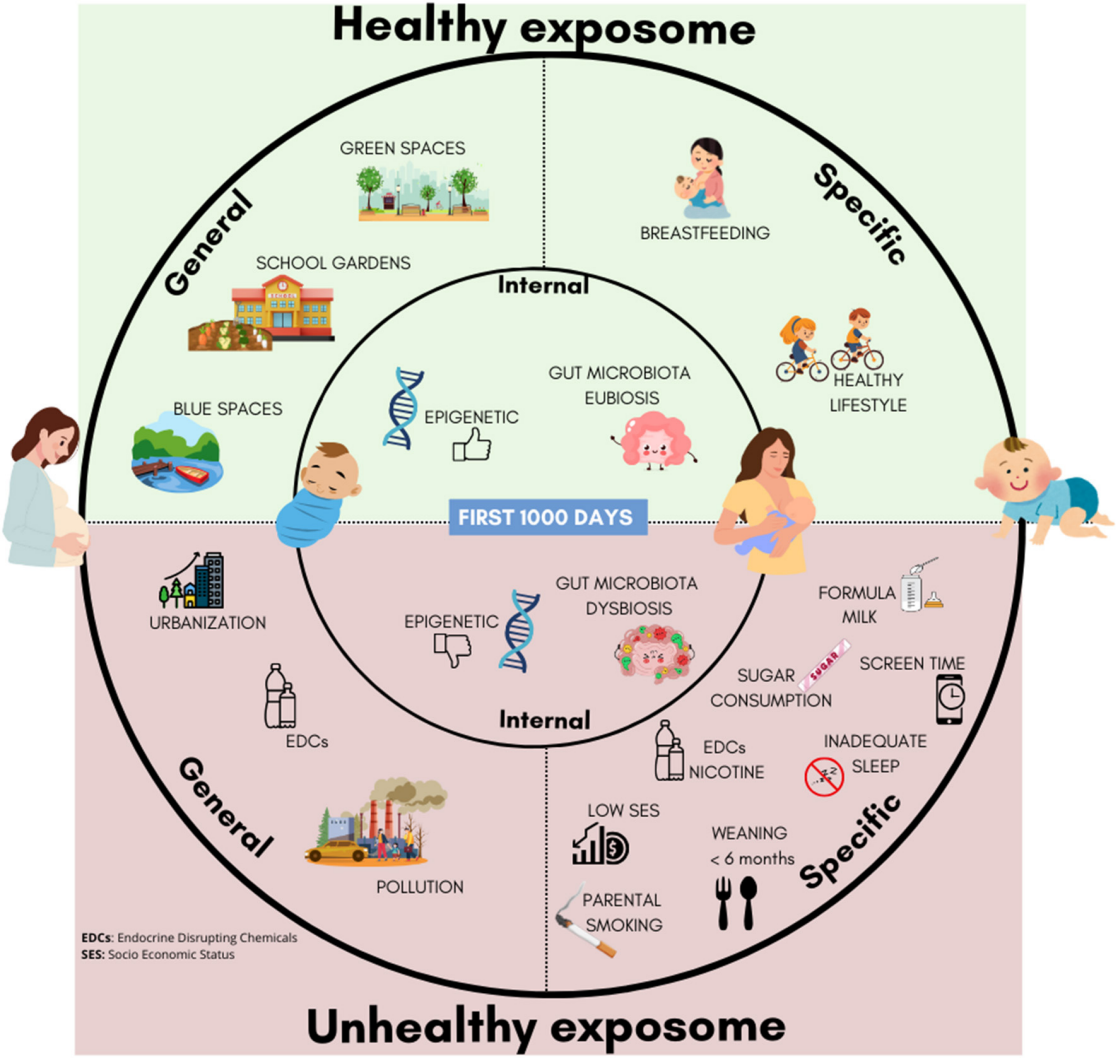
Healthy and unhealthy esposome factors in early life: general, specific, and internal determinants influencing childhood obesity risk.

According to the literature findings, the authors identified a “healthy exposome,” encompassing protective factors against obesity, and an “unhealthy exposome” associated with higher obesity risk (Figure 1).

Clear evidence of protective factors emerged across different domains of the exposome (33–35). Within the general external exposome, results indicate that access to GABS protects against obesity by supporting healthier growth trajectories, increased PA, and healthier dietary patterns (33–35).

Similarly, regarding the specific external exposome, exclusive breastfeeding for at least 6 months, adequate complementary feeding, regular PA, limited screen time, and proper sleep represent robust protective factors (64, 65, 85–88). These exposures act through internal pathways, including modulation of gut microbiota composition, regulation of AR, and epigenetic effects on obesity-related genes (110–112, 117, 130).

In contrast, evidence indicates that key risk factors include maternal smoking during pregnancy and early-life exposure to EDCs, such as BPA and phthalates, which have been shown to promote obesogenic processes by disrupting adipogenesis, altering glucose and lipid metabolism, and enhancing proinflammatory signaling (103, 107–110, 134).

However, the role of several factors remains controversial, including evidence regarding SES and the exposure to PFAS, for which findings are heterogeneous and context-dependent (17, 99). Moreover, although breastfeeding is consistently protective, conflicting evidence exists regarding its biochemical composition. For instance, Vieira Queiroz De Paula et al. reported heterogeneous associations of hormones (e.g., leptin, adiponectin, insulin) and macronutrients with later obesity risk (72). These discrepancies highlight the complexity of exposome research and the need for more longitudinal cohort studies.

Addressing these exposures early in life is essential, as they may have compounding effects that influence health outcomes and potentially persist across generations (9, 138, 139).

**Figure 2:**
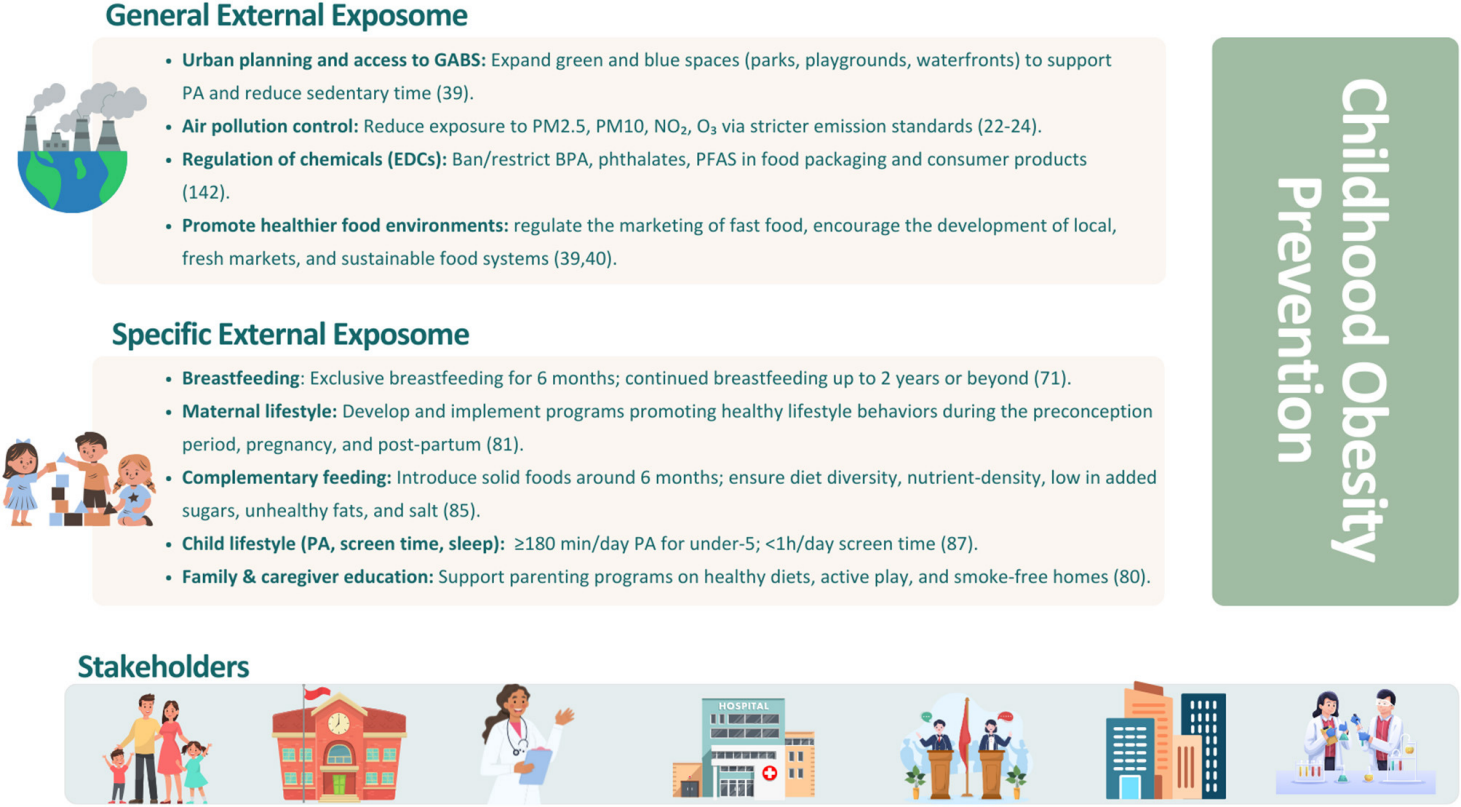
Summary of public health interventions targeting exposome domains to foster healthy early-life environments and behaviors, reducing childhood obesity risk.

Furthermore, One Health challenges, such as biodiversity loss, climate change, and ecosystem degradation, lead to exacerbation of exposure to air pollution, EDCs, and unhealthy food environments, thereby contributing to obesity and NCDs (140). To address these challenges, integrated actions are needed. Accordingly, the authors present a roadmap of actions aimed at promoting a healthy exposome during the first 1,000 days of life (Figure 2).

Accordingly, recent regulatory actions, including the 2023 EFSA safety assessment that reduced the tolerable daily intake of BPA (from 4 μg/kg bw/day to 0.2 ng/kg bw/day) and the 2024 European Commission ban on its use in food contact materials, represent key steps forward in limiting early-life exposure to these chemicals (141).

In addition, the WHO highlights six priority areas for action, such as promotion of healthy diets, increased PA, preconception and pregnancy care, early childhood nutrition, school-based interventions, and weight management, highlighting that only an integrated strategy can effectively address the modifiable risk factors identified and reduce the global burden of childhood obesity (142).

Despite the innovative approach adopted, this review has certain limitations that must be acknowledged. Primarily, not all factors of the general and specific external exposome were analyzed. This limitation arises from the inherent difficulties in assessing the complex and multifactorial nature of environmental exposures during the critical “first 1,000 days”. Second, while a range of exposures were considered, not all were assessed within the prenatal period, with some factors being investigated exclusively during early childhood. Furthermore, the results concerning certain exposome components were inconsistent, which can largely be attributed to the heterogeneity of the available research. Variations in study designs, population characteristics, and exposure assessment methodologies contribute to these discrepancies, thereby limiting the ability to generalize some of the conclusions derived from the findings.

## 6 Conclusion

During the first 1,000 days of life, general and specific external exposures critically shape childhood obesity risk through their effects on internal biological processes. A functional exposome approach, integrating environmental, behavioral, and biological data, allows the identification of critical windows of vulnerability and supports early interventions that limit harmful exposures. Interdisciplinary collaboration is essential to unravel the complex interplay between genetic susceptibility, environmental determinants, and lifestyle-related factors, thereby enabling the development of tailored prevention strategies. Future research should prioritize the integration of big data analytics, machine learning, and epidemiological studies to clarify inconsistent findings and uncover exposure patterns not detectable with traditional methods. This approach enhances the ability to design evidence-based policies, regulatory frameworks, and community-based initiatives that reduce disease prevalence and improve overall public health outcomes.
